# Instrumentation and Automated Photometric Titration Procedure for Total Acidity Determination in Red Wine Employing a Multicommuted Flow System

**DOI:** 10.1155/JAMMC/2006/83247

**Published:** 2006

**Authors:** Ausberta Jesus Cabezas Garcia, Boaventura F. Reis

**Affiliations:** 1 Instituto de Química de São Carlos Universidade de São Paulo Avenue Centenário 303 P.O. Box 96, Piracicaba São Paulo SP CEP 13400-970 Brazil; 2 Centro de Energia Nuclear na Agricultura Universidade de São Paulo Avenue Centenário 303 P.O. Box 96, Piracicaba São Paulo SP CEP 13400-970 Brazil

## Abstract

An automated procedure for photometric titration of red wine and associated instrumentation is described. The procedure was based on the flow-batch approach implemented employing multicommutation. The photometric detection was carried out using a homemade LED-based photometer. The mixing device, LED, and photodetector were attached to the titration chamber in order to form a compact and small-sized unit. The flow system comprised an automatic injector and three-way solenoid valves, which were controlled by a microcomputer through an electronic interface card. The software, written in Quick BASIC 4.5, was designed with abilities to accomplish all steps of the titration procedure including data acquisition and real-time processing to decide about the course of titration in the following step and so forth, until the titration endpoint was reached. The usefulness of the proposed titration system was demonstrated by analyzing red wine samples. When results were compared with those obtained using the AOAC reference method, no significant difference was observed at the 95% confidence level. A relative standard deviation of *ca*  2%  (n=9) was obtained when processing a typical red wine sample containing 7.3 ${\text{gl}}^{-1}$ total acidity expressed as tartaric acid.


Hindawi Publishing Corporation
Journal of Automated Methods and Management in Chemistry
Volume 2006, Article ID 83247, Pages 1–8
DOI 10.1155/JAMMC/2006/83247

### Instrumentation and Automated Photometric Titration Procedure for Total Acidity Determination in Red Wine Employing a Multicommuted Flow System

**Ausberta Jesus Cabezas Garcia[1] and Boaventura F. Reis[2]**

[1] *Instituto de Química de São Carlos, Universidade de São Paulo, Avenue Centenário 303, P.O. Box 96, Piracicaba, CEP 13400-970 São Paulo, SP, Brazil*
[2] *Centro de Energia Nuclear na Agricultura, Universidade de São Paulo, Avenue Centenário 303, P.O. Box 96, Piracicaba, CEP 13400-970 São Paulo, SP, Brazil*

Received 26 April 2006; Revised 28 August 2006; Accepted 6 September 2006

An automated procedure for photometric titration of red wine and associated instrumentation is described. The procedure was based on the flow-batch approach implemented employing multicommutation. The photometric detection was carried out using a homemade LED-based photometer. The mixing device, LED, and photodetector were attached to the titration chamber in order to form a compact and small-sized unit. The flow system comprised an automatic injector and three-way solenoid valves, which were controlled by a microcomputer through an electronic interface card. The software, written in Quick BASIC 4.5, was designed with abilities to accomplish all steps of the titration procedure including data acquisition and real-time processing to decide about the course of titration in the following step and so forth, until the titration endpoint was reached. The usefulness of the proposed titration system was demonstrated by analyzing red wine samples. When results were compared with those obtained using the AOAC reference method, no significant difference was observed at the 95% confidence level. A relative standard deviation of *ca* 2% ($n = 9$) was obtained when processing a typical red wine sample containing 7.3 $gl^{-1}$ total acidity expressed as tartaric acid.

Copyright © 2006 A. J. C. Garcia and B. F. Reis. This is an open access article distributed under the Creative Commons Attribution License, which permits unrestricted use, distribution, and reproduction in any medium, provided the original work is properly cited.

### 1. INTRODUCTION

In general, the acids present in wines are formed during the fermentation process and tartaric acid is the major constituent [1]. Total acidity is related to the main enological parameters as reported by several authors [2–5]; nevertheless, volatile acidity is also a parameter used to evaluate wine and acetic acid is considered its main component [6, 7]. Studies involving speciation model [8], astringency properties [9], and vineyard irrigation [10] have been elected as relevant parameters for total wine acidy. In this sense, the availability of a reliable procedure for acidity determination is mandatory. Acid-base titration is a widely used methodology for total acidity determination in wine, using an external indicator solution [11–13], although procedures based on voltammetry and conductometry have also been proposed [14, 15].

Automated titration has greatly evolved by employing flow injection analysis process to handle solutions and detection by spectrophotometry [16–19]. The strategies adopted allowed titration endpoint detection; nevertheless, acidity quantification was dependent on an analytical curve, which was derived from measurements obtained by processing a set of standard solutions. This condition was not required when using the multicommuted process in the flow system [20–22], thus allowing true titration to be implemented for the first time [23, 24], which was done by exploiting a binary search strategy. Using the means provided by binary search strategy, a procedure was developed to determine acidity in silage material, with the ability to compensate the effect caused by sample color [25]. The binary sampling strategy was also employed to implement true titration procedures using potentiometry as a detection technique [26]. The ease afforded by the multicommutation process to handle solutions allowed ionic strength to be maintained in the bulk sample, since this condition is a mandatory requirement in potentiometric titration [24]. Honorato et al. developed an automatic spectrophotometric titration procedure employing the flow-batch approach [13]. The strategy to find the titration endpoint was based on Fibonacci's method, which was implemented by means of multicommutation process.

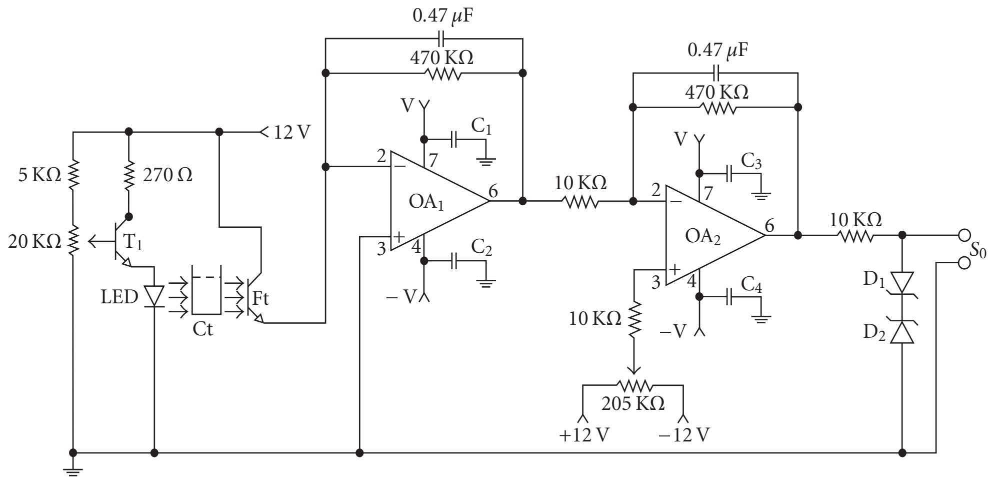

Figure 1: *Electronic diagram of the photometer*. $T_1$= transistor (BC547); LED = light emitting diode, $\lambda$ = 545 nm; Ct = titration chamber; Ft = phototransistor Til78; $OA_1$ and $OA_2$ = operational amplifier (OP07); $D_1$ and $D_2$ = Zenner diode, 4.7 V; $S_0$ = output signal; $C_1$, $C_2$, $C_3$, $C_4$ = tantalum capacitors, 2 $\mu$F. The power supply used to feel this circuit provided stabilized difference of potential (+12 V, −12 V) and low level of noise ≅ 3 mV.

The procedure allowed the quantifying of the sample acidity without using an analytical curve. The feasibility of the procedure was demonstrated by carrying out total acidity determination in white wine.

Red wines absorb electromagnetic radiation in a wide range of the visible spectrum. Therefore, this feature can make titration endpoint detection difficult when an external indicator such as phenolphthalein is used in photometric detection. Aiming to overcome this drawback, in this work we intend to design an integrated setup comprising the titration module and the electronic device for photometric detection, which will be employed to develop an automatic procedure for total acidity determination in red wine. The integrated setup will be designed to implement the titration procedure based on the flow-batch approach [13]. The control software will be developed with abilities to recognize the titration endpoint using phenolphthalein as external indicator. Attention will be paid to construct a small-sized equipment without sacrificing the overall performance of the analytical procedure.

### 2. EXPERIMENTAL

### 2.1. Solutions

Fresh deionized water boiled for 15 min to remove carbon dioxide was used as carrier fluid and to prepare titration solutions. Titration solutions at concentrations of 0.05 and 0.10 $mol^{-1}OH^-$ were prepared by dilution with water from a 10 $mol^{-1}NaOH^-$ stock solution. A standardized 0.0092 $mol^{-1}OH^-$ solution was prepared by taking a portion of the supernatant NaOH stock solution that was diluted with water and standardized using a potassium biphthalate solution. This solution was prepared before use and stored in a polyethylene bottle. A 0.01% (w/v) phenolphthalein solution was prepared daily by diluting with ethanol from a 1% (w/v) stock solution prepared in ethanol medium.

Red wine samples were purchased from the local market. Before analysis, 50 mL aliquots were transferred to the working bottles and an argon stream was bubbled for 20 min in order to remove dissolved carbon dioxide. These samples were also processed employing the AOAC reference method [12]. After the $CO_2$ removal step, solutions and wine samples could be used for a period of four hours.

### 2.2. Apparatus

The equipment setup comprised a microcomputer furnished with an electronic interface card PCL711S (American Advantech Corp.); an IPC8 Ismatec peristaltic pump equipped with Viton pumping tubes; four three-way solenoid valves (Nresearch, 161T031); an automatic injector [27]; a regulated 12 V power supply to feed the solenoid valves, mixing device, and solid state relay; and flow line of Teflon tubing 0.8 mm inner diameter. The electronic interface control to drive the injector and solenoid valves based on the integrated circuit ULN2803 was similar to that employed in earlier works [28, 29]. The homemade photometer tailored to implement the titration procedure is described below.

### 2.3. The photometer

The titration procedure used phenolphthalein as an external indicator, thus a green LED with maximum emission intensity at 545 nm was employed as light source and a phototransistor (Til78) was used as detector. The electronic circuitry required for signal generation and its amplification is shown in Figure 1. The first operational amplifier ($OA_1$) and the phototransistor (Ft) comprised the signal-generating network, which converted light coming from the LED into electric potential difference. The second operational amplifier ($OA_2$) was configured to provide five-fold signal amplification and to allow base line adjustment.

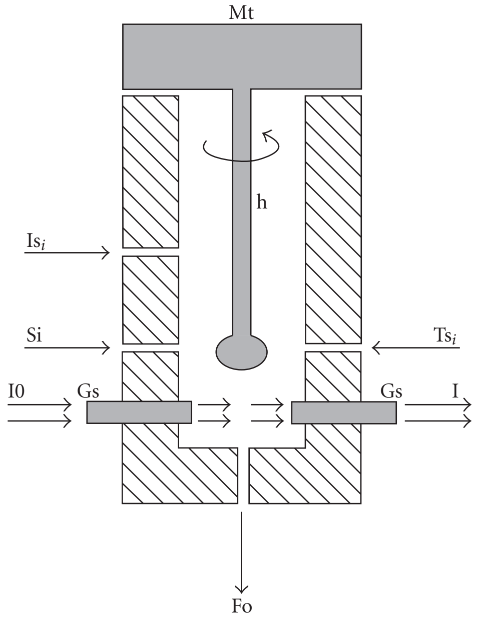

FIGURE 2: *Pictorial view of the titration chamber*. The hatched surface represents a longitudinal cut view of the titration chamber. Mt = motor; h = Teflon mixing stem, 30 mm long and 4 mm diameter; $Is_i$, Si, and $Ts_i$ = input for external indicator solution, sample, and titrant solution, respectively; Gs = glass cylinder, 30 mm long and 3.0 mm diameter, $I_0$ and I = radiation beams coming from the radiation source (LED) and after crossing the titration chamber, respectively; Fo = hole for solution draining.

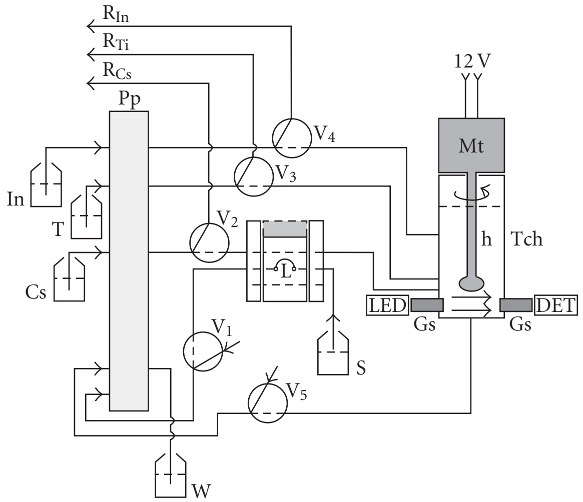

FIGURE 3: *Flow diagram of the titration system*. The three rectangular surfaces are an overview of the automatic injector, shadow surface indicates the alternative position of the sliding bar (central part); Cs = carrier fluid (water) flow rate at 30.0 $\mu Ls^{-1}$; T = titrant solution (NaOH), flow rate at 10.4 $\mu Ls^{-1}$; In = indicator solution (phenolphthalein), flow rate at 10 $\mu Ls^{-1}$; S = sample (red wine), flow rate at 10 $\mu Ls^{-1}$; W = waste, flow rate at 64 $\mu Ls^{-1}$; Pp = peristaltic pump; $V_1, V_2, \ldots, V_5$ = three-way solenoid valves; L = sampling loop, 16.0 $\mu$L inner volume; Gs = glass cylinder; LED = light emitting diode, $\lambda$ = 545 nm; DET = phototransistor (Til78); Tch = titration chamber, h = mixing stem. Solid lines and dashed lines in the valves symbols indicate the fluid pathway when valves are switched OFF or ON, respectively. Arrows indicate the pumping direction.

### 2.4. *Titration chamber*

The titration chamber, with a cylindrical geometry, was machined in a Teflon block, which comprised a cylindrical hole perforated at the longitudinal axis of the Teflon block with 10 mm inner diameter and 40 mm height, totaling an inner volume of 3.14 mL. A pictorial representation of the titration chamber is shown in Figure 2. The titration chamber was designed to allow direct signal measurement; consequently, and to attain this requirement two glass cylinders (Gs) were installed to work as a waveguide. To avoid fluid leakage, the glass cylinders were embedded using a rubber gasket. The first glass cylinder transmits the radiation beam ($I_0$) coming from a light source through the titration chamber wall, while the second collects the light beam (I) after crossing the solution into the titration chamber and sends it towards a photodetector. To obtain an effective mixing condition a small direct current motor (Mt) was installed on top of the titration chamber that was attached with a screw to the cap, which was controlled by the microcomputer through the control interface [20].

A cylindrical Teflon piece (30 mm length, 4 mm diameter) with a flat tip was attached to the motor axis. Its length was adjusted to maintain the flat tip 3 mm above the light beam.

The roller counter output available from the IPC-8 Ismatec peristaltic pump was attached to the $A_1$ analog input of the PCL711S interface card by means of a shielded cable. This attachment was designed to allow time synchronization between pumping pulsation and the valve switching step to insert the titrant solution into the titration chamber.

### 2.5. *The titration system*

The flow diagram of the titration system is depicted in Figure 3. In this configuration, all valves are switched OFF, thus carrier fluid (Cs), titration solution (T), and indicator solution (In) are pumped back to their storage vessels $R_{Cs}$, $R_{Ti}$, $R_{In}$, respectively, while the sample stream (S) was stopped. The displacement of the injector sliding bar (central part) from the sampling position to the insertion position and vice versa was performed by means of two solenoids attached to the central sliding bar [27], which were not shown to simplify the diagram. Injector displacement was controlled by the microcomputer sending electric pulses through the control interface.

When the software was run, the microcomputer maintained valves $V_1$, $V_2$, $V_3$, and $V_4$ switched ON during a time interval of 30 s in order to fill the flow lines with the respective solutions. Next, valve $V_5$ was switched ON for a time interval of 45 s to empty the titration chamber (Tch), which was then washed with carrier solution (Cs) and emptied

Table 1: Steps to collect reference signal without using the day indicator.

| Step | $V_1$ | $V_2$ | $V_3$ | $V_4$ | $V_5$ | Isp[†] | Iip | Motor | Time (s) |
|---|---|---|---|---|---|---|---|---|---|
| Injector displacing to sampling position ($St_0$) | OFF | OFF | OFF | OFF | OFF | ON | OFF | OFF | 1 |
| Loop loading ($St_1$) | ON | OFF | OFF | OFF | OFF | OFF | OFF | OFF | 20 |
| Injector displacing to inserting position ($St_2$) | OFF | OFF | OFF | OFF | OFF | OFF | ON | OFF | 1 |
| Sample inserting ($St_3$) | OFF | ON | OFF | OFF | OFF | OFF | OFF | OFF | 20 |
| Titrant solution adding ($St_4$)* | OFF | OFF | ON | OFF | OFF | OFF | OFF | OFF | 10 |
| Mixing solutions ($St_5$)* | OFF | OFF | OFF | OFF | OFF | OFF | OFF | ON | 5 |
| Signal reading ($St_6$)* | OFF | OFF | OFF | OFF | OFF | OFF | OFF | OFF | 5 |
| Compare titrand volume ($St_7$)* | OFF | OFF | OFF | OFF | OFF | OFF | OFF | OFF | — |
| Chamber emptying ($St_8$) | OFF | OFF | OFF | OFF | ON | OFF | OFF | OFF | 20 |
| Chamber washing ($St_9$) | OFF | ON | OFF | OFF | OFF | OFF | OFF | OFF | 35 |
| Chamber emptying ($St_{10}$) | OFF | OFF | OFF | OFF | ON | OFF | OFF | OFF | 20 |

[†]Isp and Iip injector sliding bar in sampling and insertion positions, respectively.
*Steps repeated up to attain the preset volume of the titrant solution.

again. This step was accomplished by sequentially switching valves $V_2$ and $V_5$ during a time interval of 20 s.

Photometer calibration was performed prior to beginning the titration run, comprising the following steps: a 600 $\mu$L aliquot of the carrier solution (Cs) was injected into the titration chamber (Tch) by switching valve $V_2$ for a time interval of 20 s. By maintaining the LED (Figure 1) switched OFF, the reading in the absence of light was adjusted to 50 mV ($OA_2$ output) through the variable resistor (20 k$\Omega$) wired to the noninverting input of the operational amplifier. Next, the LED emission intensity was increased until the output measurement reached 1800 mV. This step was done by turning forward the variable resistor attached to the base of the transistor ($T_1$). After the photometer was calibrated, the chamber was emptied by switching ON the $V_5$ valve.

After running for a period of four hours, the calibration step could be carried out again. It was observed that at constant temperature, the dark measurement (50 mV) and calibration signal (1800 mV) presented no significant variations.

After the photometer was calibrated, the microcomputer carried out the titration run by performing the sequence of events summarized in Table 1. The sliding bar of the injector (Figure 3) was switched to the loop loading position ($St_1$) to fill the sampling loop (L) with a wine aliquot. Then, the sliding bar was switched to the insertion position (shaded surface). As indicated in Figure 3, in this position the sampling loop (L) was placed in the pathway of the carrier fluid (Cs). Valve $V_2$ was switched ON for 20 s (step $St_3$) in order to displace the sample aliquot from the sampling loop (L) to the titration chamber (Tch); therefore, adding 600 $\mu$L of carrier fluid. Next, the titrant solution addition step ($St_4$) was carried out by switching valve $V_3$. After the solution was homogenized (step $St_5$), the signal generated was read (step $St_6$) by the microcomputer through the analog input ($A_0$) of the PCL711s interface card. The measurement was saved as an ASCII file to allow further treatment. The comparison step ($St_7$) verifies if the titration solution volume (T) injected into the titration chamber reached a preset value (1000 $\mu$L). Steps $St_4$, $St_5$, $St_6$, and $St_7$ were repeated until this condition was attained. Afterwards, the titration chamber was drained and cleaned by performing steps $St_8$, $St_9$, and $St_{10}$.

The titration run described above was carried out without adding the indicator solution to the titration chamber. Under this condition, the signal detected was due to red wine absorption. As indicated in the previous paragraph, the photometer was adjusted to generate an output signal ($S_0$) of 1800 mV with the titration chamber filled with water. Under this condition, the phototransistor received the maximum light intensity. In this sense, when a wine sample was processed, the output signal (WineSignal) was lower than 1800 mV, and the analytical signal (AnSignal) was the difference between these measurements (AnSignal = 1800-WineSignal). After the titration run described above was completed, the software selected the measurement with the highest value, which was adopted as a reference signal (RefSignal = $\text{AnSignal}_{max}$). This reference signal was used to decide when the titration runs should be finished, which were implemented using an endpoint indicator solution (phenolphthalein) following the sequence depicted in Table 2.

The related steps from $St_0$ to $St_7$ in Table 2 were performed as described for identical steps in Table 1. In the $St_8$ step the highest signal (AnSignal) achieved in step $St_7$ was compared against the reference measurement (RefSignal) selected, as described in the last paragraph. The comparison between AnSignal and RefSignal was performed to make a decision with regard to the next step of the titration run,

Table 2: Steps followed to perform a titration run.

| Step | $V_1$ | $V_2$ | $V_3$ | $V_4$ | $V_5$ | Isp† | Iip | Motor | Time (s) |
|---|---|---|---|---|---|---|---|---|---|
| Injector displacing to sampling position ($St_0$) | OFF | OFF | OFF | OFF | OFF | ON | OFF | OFF | 1 |
| Sampling loop loading ($St_1$) | ON | OFF | OFF | OFF | OFF | OFF | OFF | OFF | 20 |
| Injector displacing to inserting position ($St_2$) | OFF | OFF | OFF | OFF | OFF | OFF | ON | OFF | 1 |
| Sample inserting ($St_3$) | OFF | ON | OFF | OFF | OFF | OFF | OFF | OFF | 20 |
| Pumping synchronization ($St_4$) | OFF | OFF | OFF | OFF | OFF | OFF | OFF | OFF | — |
| Titrant solution adding ($St_5$) | OFF | OFF | ON | OFF | OFF | OFF | OFF | OFF | [1]$T_0$ |
| Mixing solutions ($St_6$)* | OFF | OFF | OFF | OFF | OFF | OFF | OFF | ON | 5 |
| Signal reading ($St_7$) * | OFF | OFF | OFF | OFF | OFF | OFF | OFF | OFF | 10 |
| Signal comparison ($St_8$)* | OFF | OFF | OFF | OFF | OFF | OFF | OFF | OFF | — |
| Pumping synchronization ($St_9$)* | OFF | OFF | OFF | OFF | OFF | OFF | OFF | OFF | — |
| Titrant solution increment ($St_{10}$)* | OFF | OFF | ON | OFF | OFF | OFF | OFF | OFF | [2]$T_i$ |
| Compare titrant volume ($St_{11}$)* | OFF | OFF | OFF | OFF | OFF | OFF | OFF | OFF | — |
| Chamber emptying ($St_{12}$)** | OFF | OFF | OFF | OFF | OFF | OFF | OFF | ON | 30 |
| Chamber washing ($St_{13}$)** | OFF | OFF | OFF | OFF | OFF | OFF | OFF | OFF | 45 |
| Solutions mixing ($St_{14}$)** | OFF | OFF | OFF | OFF | OFF | OFF | OFF | ON | 5 |
| Chamber emptying ($St_{15}$) | OFF | OFF | OFF | OFF | ON | OFF | OFF | OFF | 30 |

†Isp and Iip injector sliding bar in sampling and insertion positions, respectively.
*Steps repeated up to the end of the titration.
**Steps repeated two times to wash the titration chamber.
[1]The time interval to insert the first aliquot of titrant solution fixed at 10.0 s;
[2]Time interval to maintain valve $V_3$ switched ON, which was defined by software as described by the equations from (1) to (4).

which was done considering the following condition:

$$\text{if AnSignal} < 0.2\text{RefSignal, then } T_i = T_0; \quad (1)$$

$$\begin{aligned} &\text{if AnSignal} \geq 0.2\text{RefSignal and AnSignal} \\ &\qquad < 0.4\text{RefSignal, then } T_i = 0.5T_0; \\ &\text{if AnSignal} \geq 0.4\text{RefSignal and AnSignal} \\ &\qquad < 0.8\text{RefSignal, then } T_i = 0.2T_0; \\ &\text{if AnSignal} \geq 0.8\text{RefSignal and AnSignal} \\ &\qquad < \text{RefSignal, then } T_i = 0.1T_0; \end{aligned} \quad (2)$$

$$\begin{aligned} &\text{if AnSignal} \geq \text{RefSignal and AnSignal} \\ &\qquad \leq (\text{RefSignal} + \text{ExtRef}), \text{then } T_i = 0.05T_0; \end{aligned} \quad (3)$$

$$\text{if AnSignal} > (\text{RefSignal} + \text{ExtRef}), \text{then the titration ends.} \quad (4)$$

The ExtRef was an external value (mV) supplied when the software was started. This parameter was used to decide when the titration run should be stopped, considering the following condition: if AnSignal > (RefSignal + Vext), then the titration run should be finished. The measurement difference corresponded to the increase was caused by the external indicator. This effect occurred when the medium became alkaline. Until this condition would not be reached, the time interval ($T_i$) after which valve $V_3$ should be switched ON was defined according to the equations depicted above. Then, steps $St_9$, $St_{10}$, $St_{11}$, $St_6$, $St_7$, and $St_8$ (Table 2) were carried out again, and this sequence was maintained. The external reference (ExtRef) was an experimental variable that was defined after some assays were carried out using a red wine sample. When the comparison carried out in step $St_{11}$ indicated that the titrant solution volume injected into the titration chamber was higher than the preset volume (1000 $\mu$L), the titration run was stopped. In this case, the software issued a message indicating that the limiting condition was surpassed without achieving the titration endpoint.

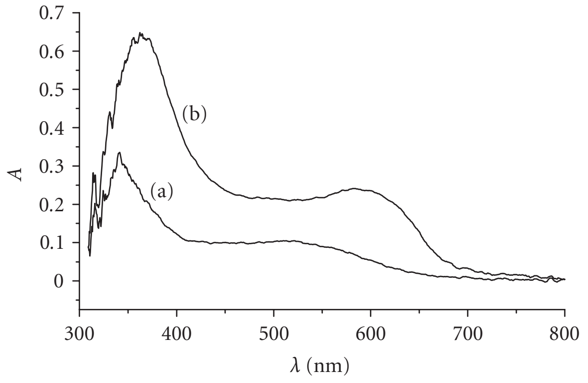

(a) pH 5
(b) pH 9.1

Figure 4: *Absorption spectra of a red wine sample.* Sample aliquots were diluted forty times with water and pH adjusted with sodium hydroxide solution.

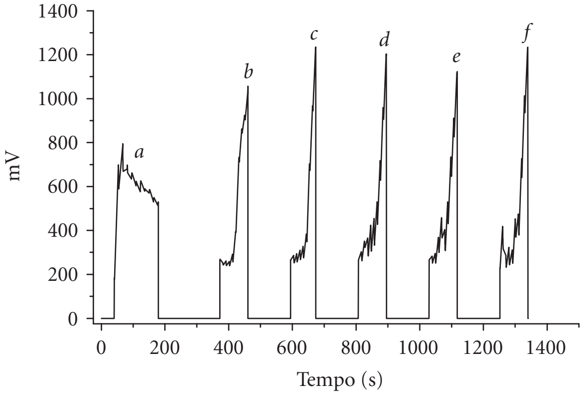

Figure 5: *Record profiles of the titration runs.* Record *a* was obtained without adding the external indicator solution to the titration chamber; other records represent titration carried out using phenolphthalein solution as indicator. Volumes ($\mu$L) of the titrant solution consumed per titration: 136.45 (*b*), 133.94 (*c*), 133.94 (*d*), 134.47 (*e*), and 134.47 (*f*). Average volume $V = (134.65 \pm 1.04)\mu$L.

The volume of the titrant solution injected into the titration chamber was controlled by the time interval elapsed while valve $V_3$ (Figure 3) was maintained switched ON. It is known that the pumping pulsation pattern could unfavorably affect the precision of the solution volume delivered [20–24]. Consequently, in order to overcome this effect, synchronization steps ($St_4$, $St_9$) were carried out prior to switching valve ($V_3$) ON, which was used to deliver the titrant solution. This task was implemented using the hardware scheme mounted by connecting the roller counter of the peristaltic pump to the analog input ($A_1$) of the PCL711S interface card. Prior to switching valve $V_3$ ON, the software execution was halted until an electric pulse coming from the peristaltic pump roller counter was detected by the microcomputer through the PCL711S interface card. Under this condition, it was assured that the valve was switched ON always at the same position of the pump roller. After the titration run ended, steps from $St_{12}$ to $St_{15}$ were carried out in order to clean the titration chamber.

Assays to find the best operational condition were carried out using titration solutions with concentrations of 0.1, 0.05, and 0.001 mol$^{-1}$OH$^-$ and processing a red wine sample. After the proper operational condition was defined, a set of red wine samples was analyzed using a standardized 0.0092 mol$^{-1}$ sodium hydroxide solution. In order to evaluate accuracy, the samples were also analyzed by the AOAC reference method [12].

### 3. RESULTS AND DISCUSSION

The records displayed in Figure 4 reveal that red wine showed significant absorption of electromagnetic radiation in the range between 350 and 700 nm, which increased with pH of the medium. This effect was probably due to the red wines, because the assays were carried out using different wine samples with similar profiles. This effect could become a drawback that must be overcome if a photometric titration procedure using phenolphthalein as an external indicator is to be used. In this sense, a strategy associating instrumentation and software was established to carry out the photometric titration procedure.

The first record shown in Figure 5 was obtained by processing an aliquot (16.0 $\mu$L) of a red wine sample without adding phenolphthalein into the titration chamber. The signal achieved when the first aliquot of the titrant solution was inserted into the titration chamber was around 250 mV. The signal increased up to 800 mV when the volume of the titrant solution added to the titration chamber was 312 $\mu$L. The signal reduction observed while the volume of titrant solution was increased until it became higher than the preset maximum volume (1000 $\mu$L) could be attributed to the sample dilution in the titration chamber. The titrant solution was added step by step, maintaining the aliquot volume at 104 $\mu$L (See Table 1, $St_5$). This volume was defined after previous assays, which were performed using different red wines and a 0.001 mol$^{-1}$ hydroxide solution. Maintaining the volume of the titrant solution aliquot at 20.8 $\mu$L and based on results obtained, it was shown that the maximum signal occurred when the titrant solution volume was within the 288 to 354 $\mu$L range. In this sense, the aliquot volume was fixed at 104 $\mu$L in order to speed up the first run, and its usefulness was demonstrated in additional assays.

Other records showed in Figure 5 were obtained by processing the titration following the conditions indicated in the experimental section (equations (1) to (3)). In accordance with the outlined titration strategy, the maximum value of measurements in the first record was selected as a reference measurement, that is, *ca* 800 mV (RefSignal = 800 mV). The external reference (4) was fixed at 100 mV, so the titration could be stopped when the signal generated by the photometer was higher than 900 mV. Taking this condition into account, the software performed the titrations represented by records labeled as *b, c, d, e*, and *f*. The records present different profiles, nevertheless the end of titration converged to

Table 3: Results comparison using the AOAC reference method. Results are presented as tartaric acid. Results are average of 5 consecutive titration runs carried out using a 0.0092 mol$^{-1}$ sodium hydroxide solution. Applying the $t$ test it was found as experimental value $t_{(95\%)} = 2.125$; theoretical value $t_{(95\%)} = 2.201$.

| Sample | Proposed procedure (g l$^{-1}$) | Reference method (g l$^{-1}$) |
|---|---|---|
| 1 | 6.39 ± 0.01 | 6.56 ± 0.24 |
| 2 | 5.70 ± 0.20 | 5.50 ± 0.20 |
| 3 | 6.50 ± 0.20 | 6.40 ± 0.20 |
| 4 | 8.50 ± 0.20 | 8.22 ± 0.09 |
| 5 | 8.11 ± 0.15 | 7.98 ± 0.09 |
| 6 | 7.08 ± 0.12 | 6.95 ± 0.05 |
| 7 | 7.11 ± 0.11 | 6.74 ± 0.12 |
| 8 | 6.04 ± 0.10 | 5.79 ± 0.07 |
| 9 | 6.48 ± 0.10 | 6.34 ± 0.04 |
| 10 | 6.15 ± 0.03 | 5.84 ± 0.04 |
| 11 | 6.10 ± 0.20 | 6.46 ± 0.07 |
| 12 | 5.95 ± 0.14 | 5.82 ± 0.08 |

Table 4: Analytical performance comparison.

| Parameters | Proposed procedure | Reference [13] |
|---|---|---|
| Wine color | Red | White |
| Concentration range (g l$^{-1}$) | 5.70–8.50 | 5.22–7.18 |
| Relative standard deviation (%) | 2% | 0.7 |
| Sample consumption per determination ($\mu$L) | 16 | 600 |
| Throughput (h$^{-1}$) | 22 | 20 |

the same volume of the titrant solution. By processing the results we obtained an average volume $V = (134.65 \pm 1.04)\,\mu$L sodium hydroxide solution, therefore, indicating that the control software was able to choose the proper course to implement the titration run. The external reference was fixed at 100 mV considering the precision of the titration solution volume, which was obtained by processing a red wine sample using 50, 100, 150, 250, and 350 mV as external references (4). In the selected case (Figure 5), the relative standard deviation was less than 1%.

Once the best operational condition was found, a set of red wine samples was analyzed in order to demonstrate the usefulness of the system, yielding the results showed in Table 3. In order to evaluate accuracy, samples were also analyzed employing the AOAC reference method [12]. By applying the paired $t$ test, no significant difference was observed at the 95% confidence level.

From the records in Figure 5 we can deduce that a time interval of 22 min was elapsed while 5 titration runs were carried out, therefore a titration throughput of 14 wine samples per hour could be expected. Nevertheless, this value could vary depending on titrant solution concentration and wine sample acidity.

Table 4 shows the overall performance of the proposed system. It can be seen that it is similar to that observed in the literature [13], which was applied for spectrophotometric titration of white wine samples.

### 4. CONCLUSION

The difficulty in detecting the photometric titration endpoint in red wine was overcome in the present work by combining instrumentation, software, and a suitable working strategy. This feature could be considered an advantage as compared to the photometric titration implemented using a titration strategy based on Fibonacci's method, which has been applied for white wine only [13].

The software ability to identify when the titration endpoint was approaching, in order to reduce the volume of titrant solution added to the titration chamber, contributed to improve the precision of the results. In this sense, we can conclude that the proper combination between instrumentation and control software is a powerful tool to implement effective analytical strategies.

Although the photometer detector was assembled using low-cost electronic components as well as very simple hardware, the performance was very good. After performing the recommended calibration, which was carried out when the photometer was switched ON, and working continuously for several hours, no significant variation in signal response was observed.

The system can be employed without any hardware or software modification for photometric titrations of other types of samples showing complex matrices.

### REFERENCES

[1] A. S. Curvelo Garcia, *Controlo de Qualidade dos Vinhos*, Instituto da Vinha e do Vinho, Lisbon, Portugal, 1988.

[2] M. Urbano-Cuadrado, M. D. Luque de Castro, P. M. Pérez-Juan, J. García-Olmo, and M. A. Gómez-Nieto, "Near infrared reflectance spectroscopy and multivariate analysis in enology. Determination or screening of fifteen parameters in different types of wines," *Analytica Chimica Acta*, vol. 527, no. 1, pp. 81–88, 2004.

[3] E. J. Bartowsky, P. J. Costello, A. Villa, and P. A. Henschke, "The chemical and sensorial effects of lysozyme addition to red and white wines over six months' cellar storage," *Australian Journal of Grape and Wine Research*, vol. 10, no. 2, pp. 143–150, 2004.

[4] S. C. Souza, K. H. Theodoro, É. R. Souza, S. Da Motta, and M. B. A. Glória, "Bioactive amines in Brazilian wines: types, levels and correlation with physico-chemical parameters," *Brazilian Archives of Biology and Technology*, vol. 48, no. 1, pp. 53–62, 2005.

[5] A. Deloire, A. Carbonneau, F. Lopez, et al., "Interaction "training system x vigour" on merlot. Comparison between vertical trellis and minimal pruning. First results," *Journal International des Sciences de la Vigne et du Vin*, vol. 38, no. 1, pp. 59–64, 2004.

[6] D. Kontkanen, D. L. Inglis, G. J. Pickering, and A. Reynolds, "Effect of yeast inoculation rate, acclimatization, and nutrient addition on icewine fermentation," *American Journal of Enology and Viticulture*, vol. 55, no. 4, pp. 363–370, 2004.

[7] D. J. Erasmus, M. Cliff, and H. J. J. Van Vuuren, "Impact of yeast strain on the production of acetic acid, glycerol, and the sensory attributes of icewine," *American Journal of Enology and Viticulture*, vol. 55, no. 4, pp. 371–378, 2004.

[8] E. Prenesti, P. G. Daniele, S. Toso, V. Zelano, and S. Berto, "Development of a speciation model for the interpretation of the acid-base properties of grape red wines," *Chemical Speciation and Bioavailability*, vol. 16, no. 1-2, pp. 17–24, 2004.

[9] G. J. Pickering, K. Simunkova, and D. DiBattista, "Intensity of taste and astringency sensations elicited by red wines is associated with sensitivity to PROP (6-n-propylthiouracil)," *Food Quality and Preference*, vol. 15, no. 2, pp. 147–154, 2004.

[10] M. Nadal and M. Lampreave, "The effects of irrigation on the water relations of the grapevine, yield, grape and wine composition of tempranillo CV in mediterranean climate," *Journal International des Sciences de la Vigne et du Vin*, vol. 38, no. 1, pp. 75–80, 2004.

[11] OIV-Office international de la Vigne et du Vin. Recueil des Méthodes Internationales d'Analyse des Vins et des Moûts, 1990.

[12] Association of Official Analytical Chemistry (AOAC), *Official Methods of Analysis*, AOAC International, Gaithersburg, Md, USA, 16th edition, 1995.

[13] R. S. Honorato, M. C. U. Araújo, R. A. C. Lima, E. A. G. Zagatto, R. A. S. Lapa, and J. L. F. C. Lima, "A flow-batch titrator exploiting a one-dimensional optimisation algorithm for end point search," *Analytica Chimica Acta*, vol. 396, no. 1, pp. 91–97, 1999.

[14] S. Ohtsuki, N. Kunimatsu, K. Takamura, and F. Kusu, "Determination of the total acid content in wine based on the voltammetric reduction of quinone," *Electroanalysis*, vol. 13, no. 5, pp. 404–407, 2001.

[15] J. Darias-Martín, A. Socas-Hernández, C. Díaz-Romero, and E. Díaz-Díaz, "Comparative study of methods for determination of titrable acidity in wine," *Journal of Food Composition and Analysis*, vol. 16, no. 5, pp. 555–562, 2003.

[16] J. Marcos, A. Ríos, and M. Valcárcel, "Automatic titrations in unsegmented flow systems based on variable flow-rate patterns. Part 1. Principles and applications to acid-base titrations," *Analytica Chimica Acta*, vol. 261, no. 1-2, pp. 489–494, 1992.

[17] J. Marcos, A. Ríos, and M. Valcárcel, "Automatic titrations in unsegmented flow systems based on variable flow-rate patterns. Part 2. Complexometric and redox titrations," *Analytica Chimica Acta*, vol. 261, no. 1-2, pp. 495–503, 1992.

[18] C. N. Yarnitzky, N. Klein, and O. Cohen, "Automated titrations with an alternate flow exponential speed variation system," *Talanta*, vol. 40, no. 12, pp. 1937–1941, 1993.

[19] E. Mataix and M. D. Luque de Castro, "Simultaneous determination of ethanol and glycerol in wines by a flow injection-pervaporation approach with in parallel photometric and fluorimetric detection," *Talanta*, vol. 51, no. 3, pp. 489–496, 2000.

[20] B. F. Reis, M. F. Gine, E. A. G. Zagatto, J. L. F. C. Lima, and R. A. Lapa, "Multicommutation in flow analysis. Part 1. Binary sampling: concepts, instrumentation and spectrophotometric determination of iron in plant digests," *Analytica Chimica Acta*, vol. 293, no. 1-2, pp. 129–138, 1994.

[21] R. N. Fernandes, B. F. Reis, and L. F. P. Campos, "Automatic flow system for simultaneous determination of iron and chromium in steel alloys employing photometers based on LEDs as radiation source," *Journal of Automated Methods and Management in Chemistry*, vol. 25, no. 1, pp. 1–5, 2003.

[22] C. K. Pires, P. B. Martelli, B. F. Reis, J. L. F. C. Lima, and M. L. M. F. S. Saraiva, "Multicommuted flow system for the determination of glucose in animal blood serum exploiting enzymatic reaction and chemiluminescence detection," *Journal of Automated Methods and Management in Chemistry*, vol. 25, no. 5, pp. 109–114, 2003.

[23] M. Korn, L. F. B. P. Gouveia, E. De Oliveira, and B. F. Reis, "Binary search in flow titration employing photometic end-point detection," *Analytica Chimica Acta*, vol. 313, no. 3, pp. 177–184, 1995.

[24] P. B. Martelli, B. F. Reis, M. Korn, and J. L. F. C. Lima, "Automatic potentiometric titration in monosegmented flow system exploiting binary search," *Analytica Chimica Acta*, vol. 387, no. 2, pp. 165–173, 1999.

[25] C. A. Tumang, A. P. S. Paim, and B. F. Reis, "Automatic flow system titration based on multicommutation for spectrophotometric determination of total acidity in silage extracts," *Journal of AOAC International*, vol. 85, no. 2, pp. 328–332, 2002.

[26] E. P. Borges, P. B. Martelli, and B. F. Reis, "Automatic stepwise potentiometric titration in a monosegmented flow system," *Mikrochimica Acta*, vol. 135, no. 3-4, pp. 179–184, 2000.

[27] P. B. Martelli, B. F. Reis, M. Korn, and I. A. Rufini, "The use of ion exchange resin for reagent immobilization and concentration in flow systems. Determination of nickel in steel alloys and iron speciation in waters," *Journal of the Brazilian Chemical Society*, vol. 8, no. 5, pp. 479–485, 1997.

[28] A. F. Lavorante, Á. Morales-Rubio, M. De La Guardia, and B. F. Reis, "Micro-pumping flow system for spectrophotometric determination of anionic surfactants in water," *Analytical and Bioanalytical Chemistry*, vol. 381, no. 6, pp. 1305–1309, 2005.

[29] G. C. Luca and B. F. Reis, "Simultaneous photometric determination of albumin and total protein in animal blood plasma employing a multicommutated flow system to carried out on line dilution and reagents solutions handling," *Spectrochimica Acta—Part A: Molecular and Biomolecular Spectroscopy*, vol. 60, no. 3, pp. 579–583, 2004.

